# Neuroprotective Effects of Black Raspberry Extract Against β‐Amyloid‐Induced Cytotoxicity in HT‐22 Cells

**DOI:** 10.1002/fsn3.70840

**Published:** 2025-09-04

**Authors:** Yohanes Tandoro, Hui‐Fang Chiu, You‐Cheng Shen, Sing‐Hua Tsou, Chih‐Li Lin, Chin‐Kun Wang

**Affiliations:** ^1^ Department of Nutrition Chung Shan Medical University Taichung Taiwan; ^2^ Faculty of Agricultural Technology Widya Mandala Catholic University Surabaya Surabaya Indonesia; ^3^ Department of Chinese Medicine Taichung Hospital Ministry of Health and Welfare Taichung Taiwan; ^4^ Department of Health Industry Management Chung Shan Medical University Taichung Taiwan; ^5^ Department of Medical Research Chung Shan Medical University Taichung Taiwan; ^6^ Institute of Medicine Chung Shan Medical University Taichung Taiwan

**Keywords:** amyloid‐β, black raspberry, oxidative stress, rutin

## Abstract

Black raspberry is known to contain a diverse number of phytochemicals, especially polyphenols which have shown health benefits. These compounds might play a role in alleviating β‐amyloid (Aβ)‐induced neurotoxicity. In this study, we investigated the effect of black raspberry in reducing Aβ toxicity and improving mitochondrial function in the HT‐22 cell model. HT‐22 cells were co‐cultured together with black raspberry extract (BRE) and Aβ and various markers for apoptosis and mitochondrial function were assessed. BRE treatment significantly improved cell viability, reduced reactive oxygen species (ROS) generation, and enhanced mitochondrial biogenesis via upregulation of PGC‐1α and Sirt1 expression. Among the isolated fractions, the ethanol fraction (Et‐F1) demonstrated the most potent neuroprotective effect. High‐performance liquid chromatography identified rutin as a major compound in Et‐F1, though it did not account for the full protective effect. In conclusion, our results suggest that BRE might be used as a natural supplementation for combatting Aβ‐associated dementia.

## Graphical abstract

This study clearly identify the role of black raspberry on neurodegeneration. After the fraction isolation, the ethanol elution showed perfect effect. The potential contributors could show mixing effect.

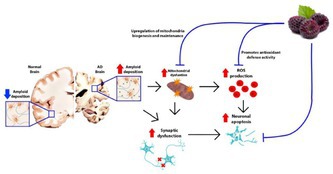

**Abbreviations**
AD: Alzheimer's disease
Aβ: amyloid‐β
BRE: black raspberry extract
CDR: clinical dementia rating
CDR: mild cognitive impairment
DAPI: 4′,6‐diamino‐2‐phenylindole
DHE: dihydroethidium
DMEM: Dulbecco's Modified Eagle's Medium
DMSO: dimethyl sulfoxide
FBS: fetal bovine serum
HPLC: high performance liquid chromatography
MTT: 3‐(4,5‐dimethylthiazol‐2‐yl)‐2,5‐diphenyltetrazolium bromide
NMDAR: N‐methyl‐D‐aspartate receptor
Nrf2: nuclear factor erythroid 2‐related factor 2
OXPHOS: oxidative phosphorylation
PGC‐1α: peroxisome proliferator‐activated receptor‐γ coactivator‐1α
PVDF: polyvinylidene difluoride
ROS: reactive oxygen species
Sirt1: Sirtuin‐1
Trk: tyrosine receptor kinases

## 1 Introduction

Neurodegenerative diseases are progressive disorders of the central nervous system characterized by irreversible neuronal damage and functional decline (Kovacs 2018). This type of disease is considered incurable due to the irreversible damage of neuronal cells. There are several types of neurodegenerative diseases, such as Parkinson's disease, Huntington's disease, and Alzheimer's disease (AD). Among them, AD is the most common type of dementia, presenting with memory loss, cognitive impairment, and behavioral disturbances that interfere with daily activities. As of 2020, over 50 million people were affected by AD worldwide, and this number is projected to double each decade (Passeri et al. 2022). Although the exact pathogenesis of AD remains unclear, one widely accepted hypothesis is the amyloid cascade, which involves the accumulation of β‐amyloid (Aβ) peptides in the brain.

Aβ mainly produced in the form of monomer, but it tends to self‐aggregate, which increases its toxicity, especially in oligomer form (Yang et al. 2022). The presence of Aβ oligomers will induce neurotoxicity, which causes neuronal cell death, and this will lead to more severe AD manifestation (Sengupta et al. 2016). Mitochondrial dysfunction is one of the results of Aβ accumulation in the brain. Neuronal cells could reuptake extracellular Aβ species and interact with cellular organelles, including mitochondria. Aβ interaction with mitochondria will result in depolarization of the mitochondrial membrane potential and increased production of ROS, which leads to increased oxidative stress and, ultimately, neuronal apoptosis (Navarro‐Hortal et al. 2024). Past studies showed that AD medication targeting Aβ did not give any meaningful improvement in both animal and human studies (Zhang et al. 2023; Panza et al. 2025). Based on the failures in previous medication experiments, further development for Aβ targeted therapy can be done to combat AD.

In recent years, increasing attention has been given to natural compounds with neuroprotective potential, especially against Aβ. Particularly, bioactive compounds like polyphenols are known to protect against neurodegeneration through multiple pathways (Arias‐Sánchez et al. 2023). A previous study showed that polyphenol‐rich blueberry powder supplementation for 12 weeks is positively improving cognitive function in middle‐aged adults with subjective cognitive decline and insulin resistance (Krikorian et al. 2022). Evidence from Marshall et al. showed that 90‐day intervention with freeze‐dried strawberry could improve several aspects of cognitive function in older adults. From the previous study, it can be concluded that daily polyphenol‐rich commodities such as berries could exert neuroprotection against cognitive decline and dementia (Miller et al. 2021; Shukitt‐Hale et al. 2015; Essa et al. 2014). Raspberry is one kind of berry fruit that belongs to the genus *Rubus* in *the Rosaceae* family (Johnson et al. 2009). Black raspberry ( *Rubus occidentalis* ) is one kind of *Rubus* berry with a unique flavor profile and health benefit potential (VanBuren et al. 2016). Black raspberry is known for its high contents of bioactive compounds such as polyphenols, especially flavonoids, anthocyanins, vitamins, and minerals, which contribute to their antioxidant, anti‐inflammatory, and anticancer activities (Kresty et al. 2016; Goodman et al. 2021; Tu et al. 2022; Zhang et al. 2022). Despite the well‐documented health effects of black raspberry, limited studies have explored its potential neuroprotective role, particularly against Aβ‐induced neuronal damage. In this study, we aimed to investigate the neuroprotective effects of black raspberry extract (BRE) in an Aβ‐induced neuronal cell damage model using HT‐22 hippocampal cells. We hypothesized that BRE could attenuate Aβ toxicity by reducing oxidative stress and improving mitochondrial function, potentially through polyphenol‐mediated modulation of key survival pathways. This research seeks to fill the existing gap in the literature regarding the role of black raspberry in neurodegeneration and to provide insights into its mechanisms of action.

## 2 Material and Methods

### 2.1 Materials

Black raspberry powder was purchased from BerryHealth (Oregon, USA). All chemicals and reagents used in this study were obtained from Sigma‐Aldrich unless otherwise specified. Primary antibodies used in this study were caspase 3, cleaved caspase 3 antibodies (CellSignaling #9662 and #9664, respectively), β‐actin (Invitrogen, # MA5‐15739), PGC‐1α (Invitrogen, # PA5‐72948), Sirt1 (Invitrogen, # MA5‐27217) and Nrf2 (Invitrogen, # PA5‐120048). Fluorescent stains, including 4′,6‐diamino‐2‐phenylindole (DAPI), Dihydroethidium (DHE), and JC‐1 were obtained from Invitrogen (catalog numbers D1306, D23107, T3168). Aβ_1‐42_ peptides (Lifetein) oligomers were prepared according to the supplier's protocol.

### 2.2 Black Raspberry Extract (BRE) Preparation

For the extraction of black raspberry polyphenols, 100 g of black raspberry powder was extracted with 300 mL of 80% aqueous ethanol for 4 h at room temperature. The mixture was then filtered to separate the solid material, and the supernatant was evaporated under vacuum at 50°C to obtain the dry BRE. The resulting dry extract was transferred to a dark‐colored bottle and stored at 4°C until further use.

### 2.3 Cell Line and Culture

Immortalized mouse hippocampal cell lines (HT‐22, Sigma‐Aldrich #SCC129) were cultured using Dulbecco's Modified Eagle's Medium (DMEM) containing 1% penicillin–streptomycin and 10% fetal bovine serum (FBS) in a humidified incubator (37°C, 5% CO_2_).

### 2.4 Cell Viability Assay

The toxicity of black raspberry extract on HT‐22 cells was determined by using 3‐(4,5‐dimethylthiazol‐2‐yl)‐2,5‐diphenyltetrazolium bromide (MTT) assay. 100 μL (0.8 × 10^5^/mL) of cells were seeded in a 96‐well plate overnight. Various concentrations of BRE (100, 250, 500, 750, and 1000 μg/mL, in DMEM), its separated fractions, or memantine (5 μg/mL) were added to the cells and incubated for 24 h. Samples were replaced with 0.5 mg/mL MTT solution (in PBS) and incubated at 37°C for 3 h. After incubation, the MTT solution was removed, 100 μL of dimethyl sulfoxide (DMSO) was added to dissolve the formazan crystal, and absorbance was measured at 570 nm using a microplate reader. Blank wells (without cells) were used as controls.

### 2.5 Neuroprotection of BRE Against Aβ‐Induced Damage

HT‐22 cells (100 μL, 0.8 × 10^5^/mL) were seeded in a 96‐well plate overnight. Various concentrations of BRE, its separated fractions, or memantine (5 μg/mL) were co‐incubated with Aβ peptides (2.5 μM) and added to the cells, then incubated for 24 h. Cell viability was measured using MTT as previously described, and relative viability was calculated by comparing treated cells with the untreated control.

### 2.6 Nuclear Fragmentation Staining by 4',6‐Diamino‐2‐Phenylindole (DAPI)

HT‐22 cells were seeded in a 24‐well plate with a concentration of 0.8 × 10^5^/mL and treated with BRE, its separated fractions, or memantine together with Aβ peptides (2.5 μM) for 16 h to induce damage but not lethal to the cells. After treatment, the cells were washed with PBS and fixed with 10% formaldehyde solution (in PBS) for 1 h. The cells were then permeabilized using ice‐cold methanol for 15 min and incubated with 0.2 μg/mL DAPI solution for 15 min. After incubation, the cells were observed using an inverted fluorescent microscope (IX‐HOS, Olympus, Japan).

### 2.7 Western Blot Analysis

HT‐22 cells were seeded in a 10 cm dish with a concentration of 0.8 × 10^5^/mL and treated with BRE, its separated fractions, or memantine together with Aβ peptides (2.5 μM). After 20 h of treatment, cells were harvested and lysed using lysis buffer. Cell proteins were collected after being centrifuged (3000 rpm) for 10 min at 4°C.

The protein was separated in 12% SDS‐polyacrylamide gels and transferred into a polyvinylidene difluoride (PVDF) membrane. 2.5% BSA solution in TBS buffer (25 mM Tris, pH 7.4, 150 mM NaCl, 0.1% Tween‐20) was used for membrane blocking. The membrane was soaked in the appropriate primary antibody and then in the appropriate HRP‐conjugated secondary antibody. The reacted protein bands were exposed by using an enhanced chemiluminescence kit (Thermo Scientific) and AI680 imaging system (GE Healthcare).

### 2.8 Mitochondrial Membrane Potential Staining by Using JC‐1

After treatment, cells were washed with PBS and incubated with a medium containing 2 μM JC‐1 (Invitrogen) for 15 min at 37°C. After incubation, the stain solution was changed to fresh medium, and cells were observed using an inverted fluorescent microscope (IX‐HOS, Olympus, Japan). Results were expressed as red and green fluorescence intensity, reflecting mitochondrial membrane potential.

### 2.9 Superoxide Detection Using Dihydroethidium (DHE) Staining

Treated cells, as mentioned in the previous staining protocol, were washed with PBS and incubated with a medium containing 10 μM DHE for 15 min at 37°C. After incubation, the stain solution was changed to a fresh medium, and cells were observed using an inverted fluorescent microscope (IX‐HOS, Olympus, Japan). Results were represented in red fluorescence intensity.

### 2.10 Polyphenol Separation From BRE

To separate the polyphenolic compounds in BRE, column chromatography was employed. A 1 g of BRE was dissolved in 2 mL of dd H_2_O and mixed for 10 min. The mixture was then poured into a separation funnel, and 2 mL of n‐hexane was added to remove lipid, oil, and non‐polar compounds. The remaining polar fraction was collected and passed through a Sephadex LH‐20 column pre‐conditioned with 95% ethanol. The column was washed with dd H_2_O before use. After the adsorption of the sample, the column was eluted with dd H_2_O, 95% ethanol, and 50% acetone at a flow rate of 1.5 mL/min. Eluted fractions were monitored at 280 nm using a UV detector for water and ethanol eluents, and at 510 nm for the acetone fraction. The solvent from each fraction was removed by vacuum evaporation, and the fractions were stored at −20°C until further use.

### 2.11 High Performance Liquid Chromatography (HPLC) Analysis

HPLC analysis was carried out to identify major components from the active fractions from BRE, as mentioned by Paudel et al. (2013). Dried extract was dissolved in acidified acetonitrile (dd H_2_O: acetonitrile: acetic acid = 69.8: 30: 0.2) and filtered using a 0.22 μm PVDF filter. Samples were analyzed by using HPLC with a binary gradient intelligent pump (L6200A, Hitachi) and a UV–vis detection system (L4250, Hitachi). A LiChrosorb (Sigma‐Aldrich, Germany) RP‐18 column (5 μm, 250 mm × 4 mm i.d.) was used to separate the sample. Aqueous 0.2% acetic acid (solvent A) and 100% acetonitrile (solvent B) were used as elution solvents by the gradient programs holding at 9% B for 10 min, 9% to 22% B from 10 to 20 min, 22% to 30% B from 20 to 35 min, 30% to 60% B from 35 to 40 min, holding at 60% B from 40 to 45 min, 60% to 9% from 45 to 50 min, and holding at 9% from 50 to 55 min. Injection volume was 50 μL and the flow rate was set at 2 mL/min. The detector was performed at 256 nm.

### 2.12 Statistical Analysis

All results were presented as the mean ± SD and the difference between treatments was assessed by SPSS (version 26, SPSS Inc.) using one‐way ANOVA and Duncan multiple comparison test. *p* < 0.05 was identified as a significant difference.

## 3 Results and Discussion

**FIGURE 1:**
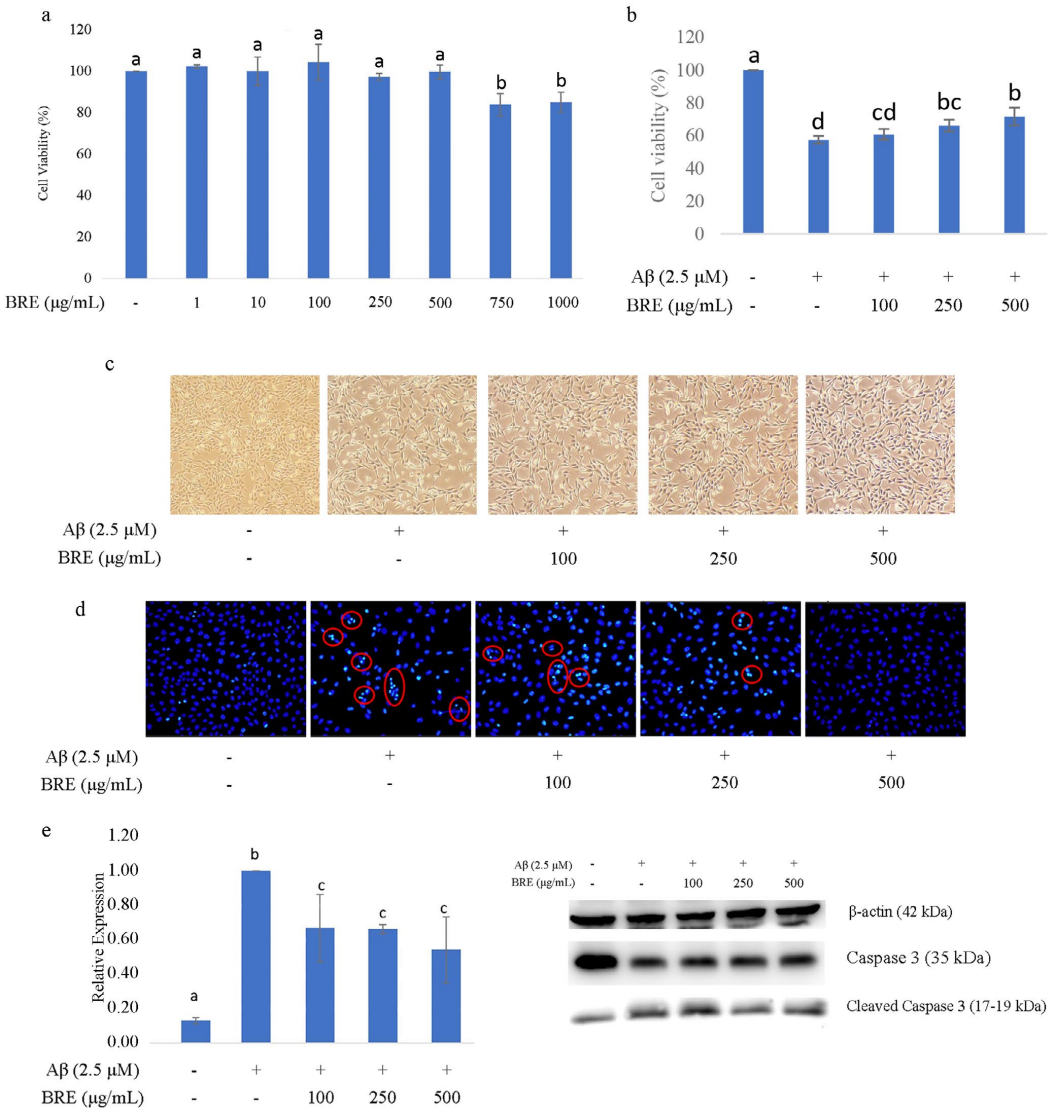
Neuroprotective effect of BRE alleviates Aβ toxicity in HT‐22 cells: (a) Cytotoxicity of BRE on HT‐22 cells; (b) BRE treatment improving HT‐22 cell viability after co‐treatment with Aβ; (c) Cell morphology after treatment; (d) BRE treatment reducing the number of fragmented nuclei shown by DAPI staining; (e) Treatment with BRE reducing apoptosis by reducing caspase 3 cleavage. Data were expressed as Mean ± SD. Different letters indicating significant difference (*p* < 0.05).

The accumulation of Aβ is a major pathogenesis of AD, leading to alterations in neuronal networks and neuronal cell death through the upregulation of apoptosis. This study focused on the suppression of Aβ toxicity by BRE using the HT‐22 cell model. 2.5 μM for Aβ concentration was chosen according to Chang et al. (2020) which demonstrated the IC_50_. The results showed that BRE treatment on HT‐22 cells up to 500 μg/mL did not exhibit any cytotoxicity (Figure 1a) and significantly improved cell viability following co‐culture with Aβ for 24 h (Figure 1b). Figure 1c showed that treatment with Aβ significantly reduced the cell numbers and induced morphological changes indicative of cell damage after 24 h of incubation. However, when co‐cultured with BRE, the damage induced by Aβ was mitigated, suggesting a neuroprotective effect of BRE. Aβ peptides, derived from amyloid precursor protein (APP) cleavage through the activity of β‐secretase and γ‐secretase, are responsible for the pathological processes in AD (Sheng et al. 2012). The accumulation of soluble Aβ oligomers, even at low concentrations, can damage neuronal cells and impair synaptic activity (Selkoe 2002; Crews and Masliah 2010).

Aβ oligomers are known to induce oxidative stress and apoptosis in neurons, a process observed in various neurodegenerative diseases including AD (Millet et al. 2005; Kumari et al. 2023). Results showed nuclear fragmentation as well as increased levels of cleaved caspase 3 after Aβ treatment, which correlated with increased apoptosis (Figure 1d,e). However, BRE treatment suppressed the apoptosis in a dose‐dependent manner, suggesting that BRE may protect neuronal cells from Aβ‐induced neurotoxicity. This protective effect is likely attributed to the polyphenols present in BRE, which exert neuromodulatory and protective effects, particularly against oxidative stress, thereby inhibiting apoptosis (Di Meo et al. 2020). Polyphenols are known to protect neuronal cells by upregulating activation of several survival pathways such as the Keap1/Nrf‐2/ARE pathway, which is responsible for antioxidant defense against oxidative stress, as well as the PI3K/Akt, PKC‐ERK1/2, and Akt‐ERK1/2 pathways, which regulate cell growth and survival (Huang and Reichardt 2003; Kim et al. 2008; Liu et al. 2016). Furthermore, polyphenols bind to tyrosine receptor kinases (Trk), activating CREB pathways, which upregulate the expression of anti‐apoptotic markers such as Bcl‐2, Bcl‐xl, as well as neurotrophic factors that inhibit apoptosis and promote neuronal survival (Huang and Reichardt 2003).

**FIGURE 2:**
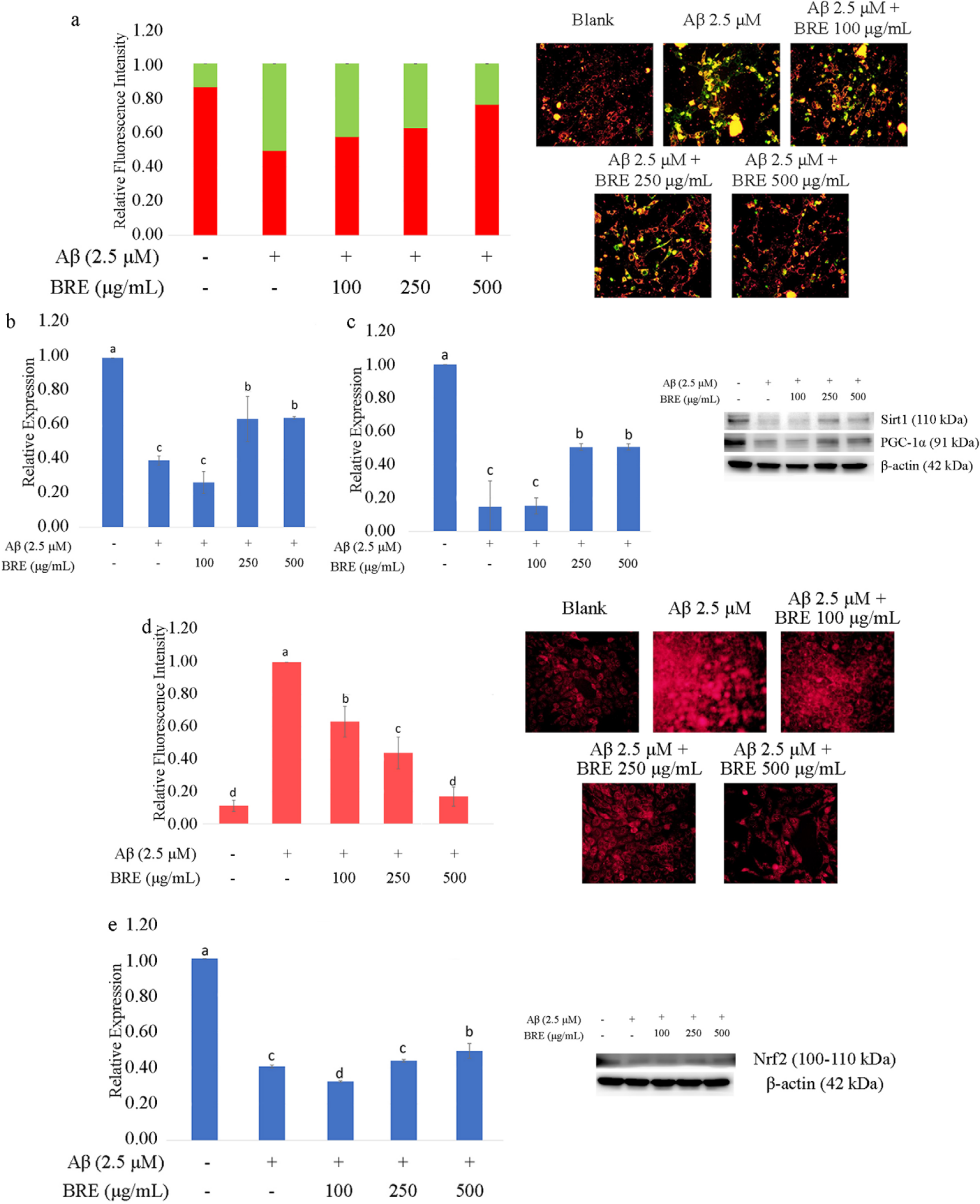
BRE treatment improving mitochondrial function of HT‐22 cells: (a) BRE treatment improving mitochondrial membrane potential shown by JC‐1 staining; Western blot analysis for expression of (b) PGC‐1α and (c) Sirt1; (d) Quantification of ROS production by DHE staining; (e) Western blot analysis for Nrf2 expression. Data were expressed as Mean ± SD, different letters indicating significant difference (*p* < 0.05).

Healthy mitochondria are essential for maintaining synaptic plasticity and neuronal function. Mitochondrial dysfunction, a hallmark of neurodegenerative diseases such as AD, can be induced by the accumulation of Aβ oligomers (Reddy and Beal 2008). Previous studies have shown that Aβ oligomers can enter cells and interact with various organelles including mitochondria. They impair mitochondrial function by disrupting mitochondrial DNA expression, as well as affecting mitochondrial biogenesis, dynamics (fusion and fission), and maintenance (mitophagy) (Reddy and Oliver 2019; Mao and Reddy 2011; Agrawal and Jha 2020; Li et al. 2024). Our findings showed that treatment with 2.5 μM Aβ induced depolarization of the mitochondria membrane in HT‐22 cells, and BRE treatment effectively prevented this mitochondrial damage, as demonstrated by JC‐1 staining in Figure 2a. Microscopic observation revealed a reduction of green and an increase in red fluorescence, indicating mitochondrial improvement. Furthermore, BRE treatment significantly upregulated sirtuin‐1 (Sirt1) and PGC‐1α expression, both of which were impaired following Aβ treatment (Figure 2b,c). PGC‐1α is known as the master regulator of mitochondrial biogenesis and function that regulates oxidative phosphorylation (OXPHOS), fatty acid metabolism, and ROS modulation (Shelbayeh et al. 2023). Qin et al. (2009) pointed out that PGC‐1α levels in the hippocampus of hyperglycemic Tg2576 AD mice and post‐mortem human brain were negatively correlated with Aβ contents and clinical dementia rating (CDR). Reduced PGC‐1α expression leads to increasing ROS generation and mitochondrial membrane potential depolarization, resulting in ROS‐induced neuronal cell death (Witte et al. 2014). Previous studies have shown that polyphenol treatment upregulates PGC‐1α expression directly or by modulating Sirt1 activation, leading to the deacetylation of PGC‐1α and increased mitochondrial biogenesis, which in turn exerts a neuroprotective effect by reducing apoptotic neurons (Zhou et al. 2018; Baur et al. 2006; Lagouge et al. 2006; Liu et al. 2015; Rehman et al. 2013).

In physiological conditions, ROS serve as signaling molecules that are essential for various biochemical processes in cells, with ROS production and scavenging tightly regulated (Zorov et al. 2014). Mitochondria are the primary source of ROS, accounting for approximately 90% of total ROS production (Balaban et al. 2005). Superoxide, a common ROS generated by the mitochondrial electron transport chain, is scavenged by antioxidant enzymes such as superoxide dismutase, maintaining a balanced ROS level within cells (Tirichen et al. 2021; Das and Roychoudhury 2014). In this study, our results clearly showed that Aβ treatment induced superoxide production and suppressed the activation of nuclear factor erythroid 2‐related factor 2 (Nrf2) (Figure 2d,e). Treatment with BRE significantly reduced intracellular superoxide and also increased the activation of Nrf2 (Ngo and Duennwald 2022). Previous studies have shown that accumulation of Aβ could result in decreasing Nrf2 signaling due to upregulation of Nrf2 suppressors such as Keap1 and GSK‐3β (Yu et al. 2015). In the early stages of AD, Aβ accumulation upregulates both Keap1 and Nrf2 levels as a protective response (Ramsey et al. 2007). However, the upregulation of Keap1 ultimately inhibits Nrf2 activity, thus reducing antioxidant defense mechanisms as well as increasing BACE1 activity, which leads to further accumulation of Aβ and decreased Nrf2 activation (De Plano et al. 2023). Polyphenols are known to directly neutralize free radicals and reduce oxidative stress, while also enhancing detoxification through the activation of Nrf2 (Scapagnini et al. 2011; Balogun et al. 2003; Romeo et al. 2009).

**FIGURE 3:**
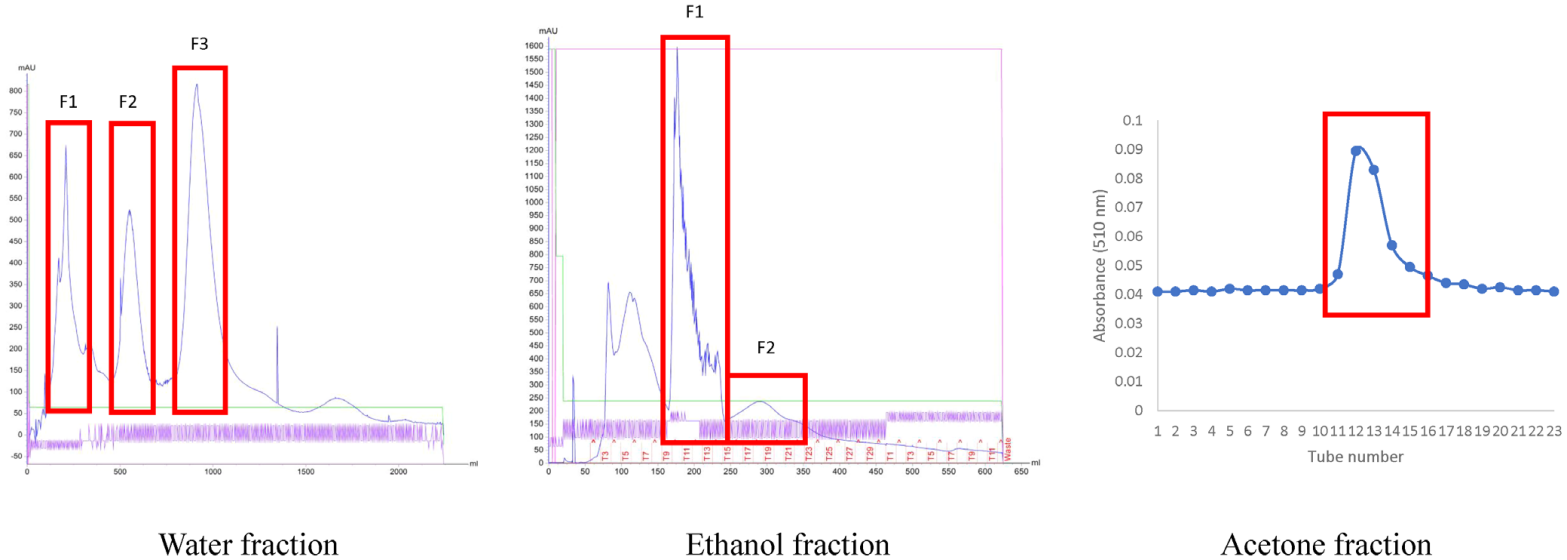
Separated BRE fractions from continuous water, ethanol, and acetone elution.

Black raspberry contains a huge variety of phytochemicals, especially anthocyanins and flavonoids (Tulio et al. 2008; Johnson et al. 2011). In this study, BRE‐separated fractions obtained from Sephadex LH‐20 chromatography were used to evaluate their effect on HT‐22 toxicity induced by Aβ. The fractions were collected according to major peaks in each solvent elution (water, 95% ethanol, and 50% acetone). All the obtained fractions, relative yield, and equivalent dose of BRE were shown in Figure 3 and tabulated in Table 1.

**TABLE 1:** BRE fraction yield.

Fraction	Fraction yield (mg)	Fraction yield (%)	Equivalent dose (μg/mL)
Water F1 (W‐F1)	1290.1	8.6	46
Water F2 (W‐F2)	58.1	0.4	2
Water F3 (W‐F3)	44.9	0.3	1.5
Ethanol F1 (Et‐F1)	120	0.8	4
Ethanol F2 (Et‐F2)	55.5	0.4	2
Acetone (Ace)	57.4	0.4	2

**FIGURE 4:**
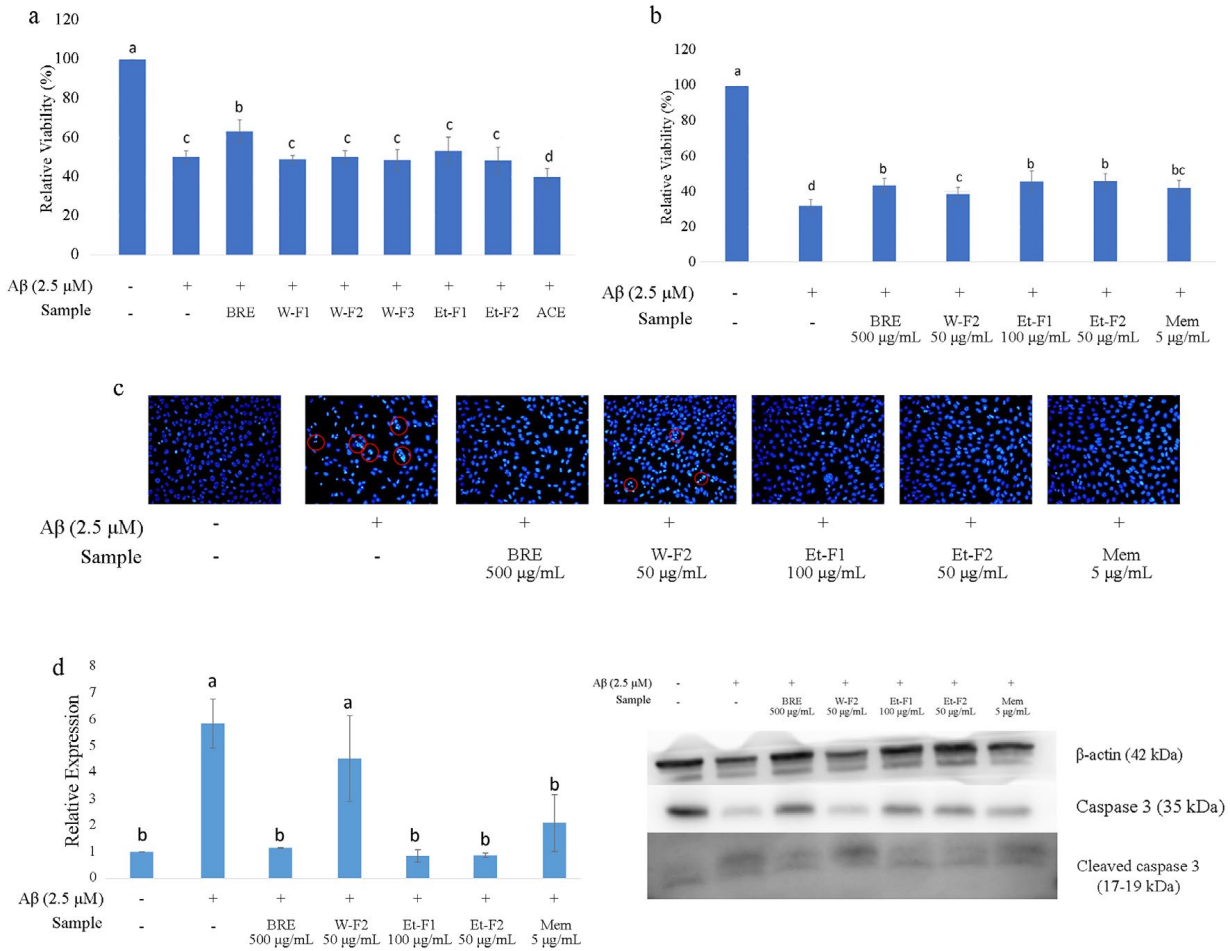
Effect of BRE and its separated fractions on the protection of HT‐22 cells by Aβ‐induced toxicity: (a) effect of equivalent dose of each fraction in BRE 500 μg/mL, (b) effect of 3 fractions at their maximum non‐toxic doses, (c) effect of separated fractions on nuclear fragmentation in HT‐22 cells after co‐culture with Aβ, (d) effect of separated fractions treatment on cleaved caspase 3 expression. Data were expressed as mean ± SD, with different letters indicating significant differences (*p* < 0.05).

Each fraction was used to evaluate its effects on Aβ‐induced damage in HT‐22 cells and compared with an equivalent dose of BRE (500 μg/mL) (Figure 4a). There was no significant improvement observed with individual fractions at their equivalent doses, suggesting that the protective effect of BRE may be a synergistic result of different phytochemicals present in the extract. Further analysis was conducted using the maximum tolerable doses for each fraction W‐F2, Et‐F1, and Et‐F2 in the concentrations of 50, 100, and 50 μg/mL, respectively. Memantine is a medication for AD, which is an N‐methyl‐D‐aspartate receptor (NMDAR) agonist that inhibits the overactivation of NMDAR in AD patients (Johnson and Kotermanski 2006). The concentrations tested for memantine (1–100 μM) showed pharmacological effects in cell culture‐based studies (Rozumna et al. 2023). In this study, 5 μg/mL (~23.1 μM) of memantine was used as a non‐toxic positive control in co‐cultures with Aβ. All treatments of BRE fractions (W‐F2, Et‐F1, and Et‐F2) showed significant improvement in cell viability. Fractions of Et‐F1 and Et‐F2 showed the highest effect (Figure 4b). Additionally, all fractions were also found to reduce nuclear fragmentation and decrease cleaved caspase 3 expression (Figure 4c,d).

**FIGURE 5:**
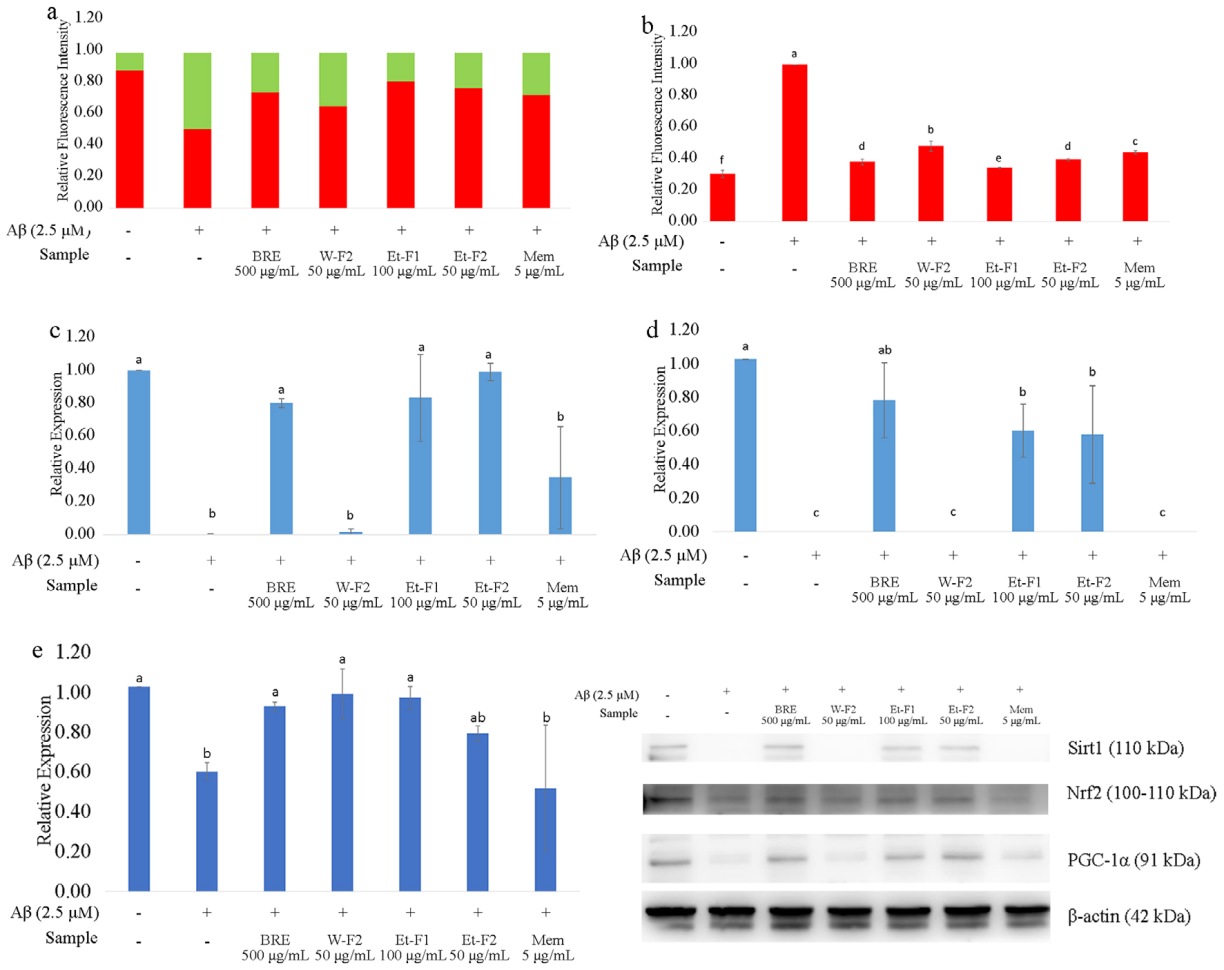
Effect of BRE separated fractions on mitochondria function: (a) Improving mitochondria membrane potential, (b) Reduction of ROS production, (c) Effect of BRE separated fractions on expression of PGC‐1α, (d) Effect of BRE separated fractions on expression of Sirt1, (e) Effect of BRE separated fractions on expression of Nrf2. Data were expressed as mean ± SD, different letters indicating significant differences (*p* < 0.05).

Fractions of Et‐F1 and Et‐F2 clearly improved mitochondrial membrane potential and reduced intracellular ROS accumulation in cells co‐cultured with Aβ (Figure 5a,b). The W‐F2 fraction also showed effects on these parameters, but it was less effective than Et‐F1 and Et‐F2. In addition, fractions Et‐F1 and Et‐F2 also showed very effective improvement in PGC‐1α, Sirt1, and Nrf2 expression (Figure 5c–e), while the W‐F2 fraction only increased Nrf2 expression without affecting PGC‐1α and Sirt1. This suggests that W‐F2 may specifically activate Nrf2, upregulating antioxidant defense without directly affecting mitochondrial maintenance function.

**FIGURE 6:**
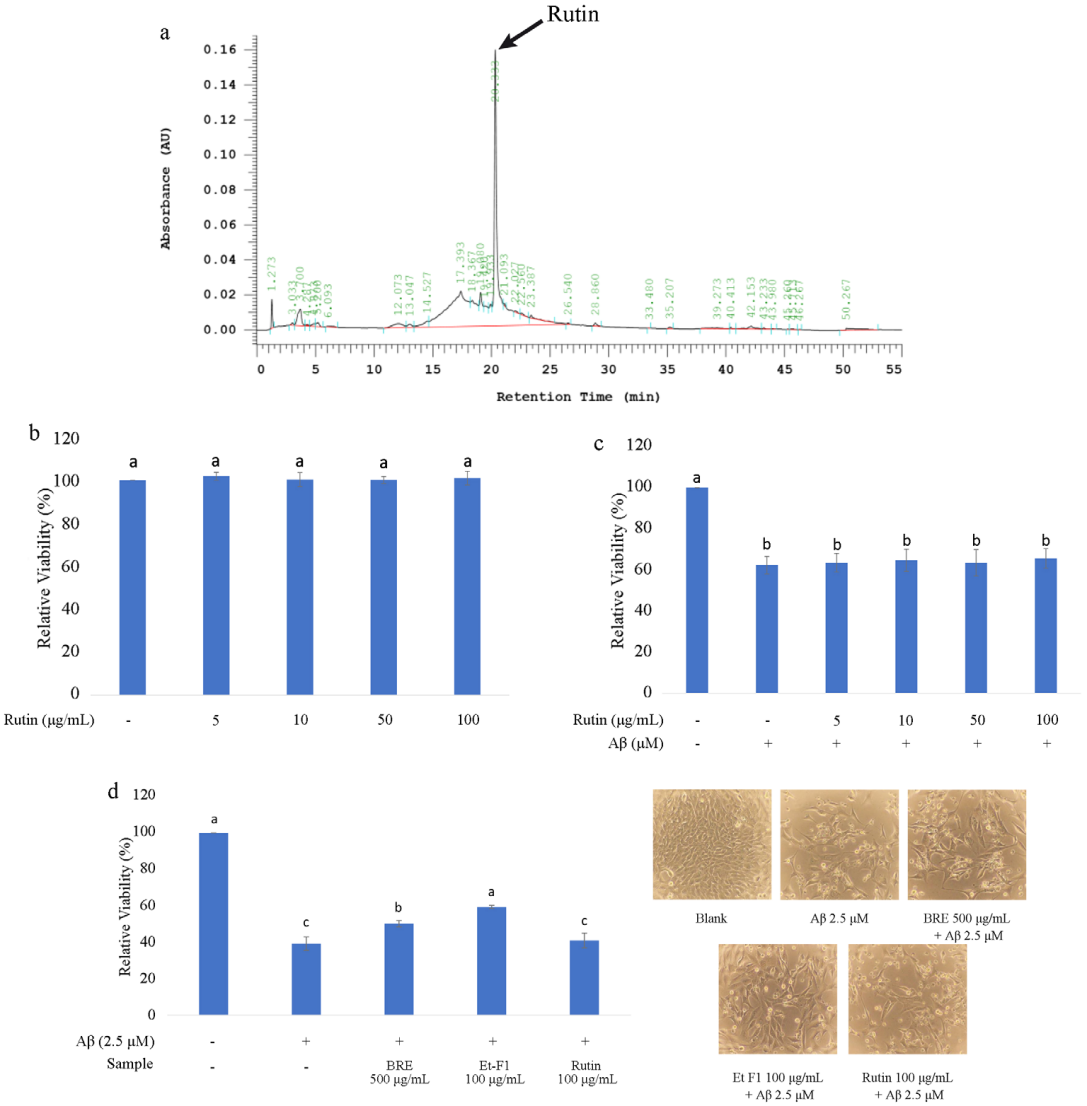
(a) HPLC analysis of Et‐F1; (b) Cytotoxicity of Rutin in various concentration; (c) Neuroprotection of various concentration of Rutin; (d) Comparison of cell viability after treatment with BRE, Et‐F1, and Rutin. Data were expressed as mean ± SD, different letter indicating significant difference (*p* < 0.05).

The Et‐F1 fraction showed significant improvement in mitochondrial function. HPLC analysis identified quercetin‐3‐rutinoside (rutin) as the major compound in Et‐F1 (Figure 6a). According to our calculation, Et‐F1 contains approximately 55.2 μg of rutin in each mg of extract, and it did not show any toxic effect to HT‐22 cells up to a concentration of 100 μg/mL, which is 20 times higher than the original concentration found in the Et‐F1 (Figure 6b). Rutin is a quercetin glycoside, and it shows pharmacological effects such as ROS scavengers and anti‐inflammatory agents (Kashyap et al. 2023). However, rutin alone did not affect cell viability when co‐incubated with Aβ (Figure 6c,d), suggesting that rutin is not the major contributor to the neuroprotective effects of Et‐F1. According to the data obtained, the main neuroprotective contributor from Et‐F1 might be a combination of several unknown compounds shown as a small peak that appears before rutin in the chromatogram (R_t_: ~17–19 min). This study showed that BRE exhibits significant neuroprotective effects against Aβ‐induced damage and modulates mitochondrial functions, with the major contributors likely residing in the Et‐F1 fraction. This result aligned with our previous pilot study where daily administration of black raspberry powder (25 g, twice daily) for 2 months could improve the CDR value of mild cognitive impairment subjects (Tandoro et al. 2025). Further studies are needed to identify other active compounds and explore the detailed mechanisms of neuroprotection, including synaptic protection.

## Author Contributions

**Yohanes Tandoro:** investigation (equal), writing – original draft (equal). **Hui‐Fang Chiu:** formal analysis (equal), resources (equal), visualization (equal). **You‐Cheng Shen:** conceptualization (equal), data curation (equal), software (equal). **Sing‐Hua Tsou:** methodology (equal), validation (equal). **Chih‐Li Lin:** investigation (supporting), methodology (supporting), resources (supporting), software (supporting). **Chin‐Kun Wang:** methodology (lead), project administration (lead), supervision (lead), writing – review and editing (lead).

## Conflicts of Interest

The authors declare no conflicts of interest.

## Supporting information


**Data S1:** fsn370840‐sup‐0001‐DataS1.pdf.

## Data Availability

The data that support the findings of this study are available on request from the corresponding author, [Chin Kun Wang]upon reasonable request.
