# Supplementary material for: Neuroprotective Effects of Black Raspberry Extract Against β‐Amyloid‐Induced Cytotoxicity in HT‐22 Cells
Source: Food Sci Nutr. 2025 Sep 4;13(9):e70840. doi: 10.1002/fsn3.70840 (PMC12411246; doi:10.1002/fsn3.70840)
Supplement: Supplementary file 1 — Data S1: fsn370840‐sup‐0001‐DataS1.pdf. [file FSN3-13-e70840-s001.pdf]

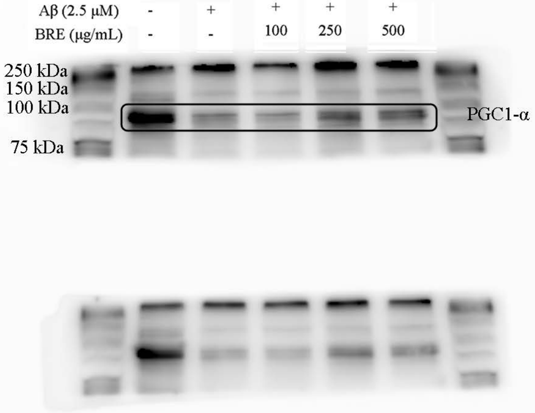
Aβ (2.5 μM) - + + + +
BRE (μg/mL) - - 100 250 500
250 kDa
150 kDa
100 kDa
75 kDa
PGC1-α

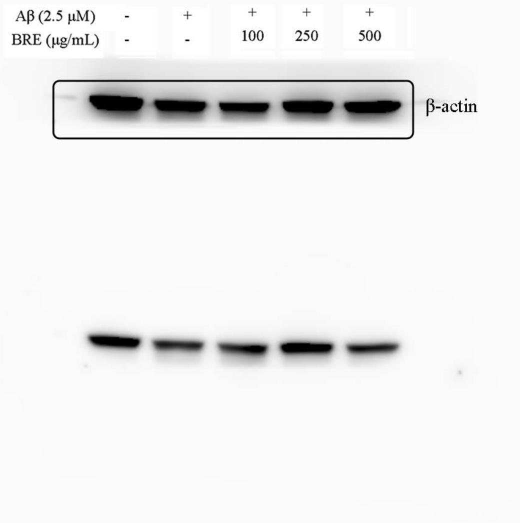

Aβ (2.5 μM) - + + + +
BRE (μg/mL) - - 100 250 500
β-actin

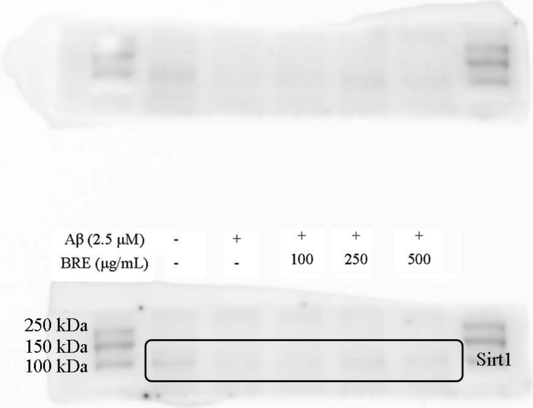
Aβ (2.5 μM)
-
+
+
+
+
BRE (μg/mL)
-
-
100
250
500
250 kDa
150 kDa
100 kDa
Sirt1

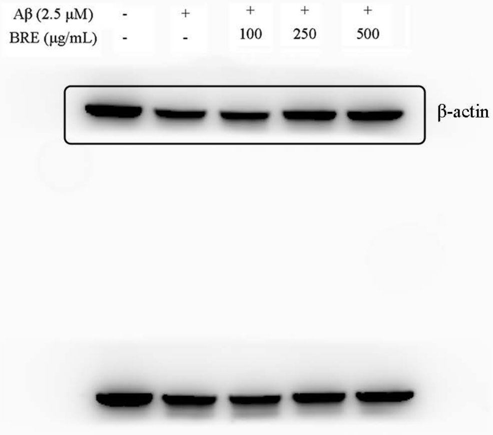

Aβ (2.5 μM) - + + + +
BRE (μg/mL) - - 100 250 500
β-actin

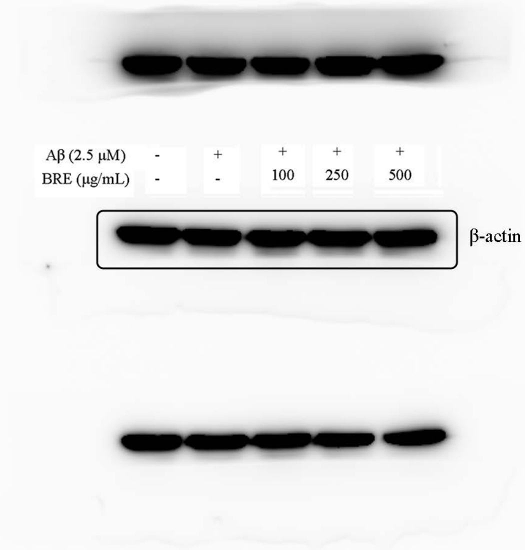

Aβ (2.5 μM) - + + + +
BRE (μg/mL) - - 100 250 500
β-actin

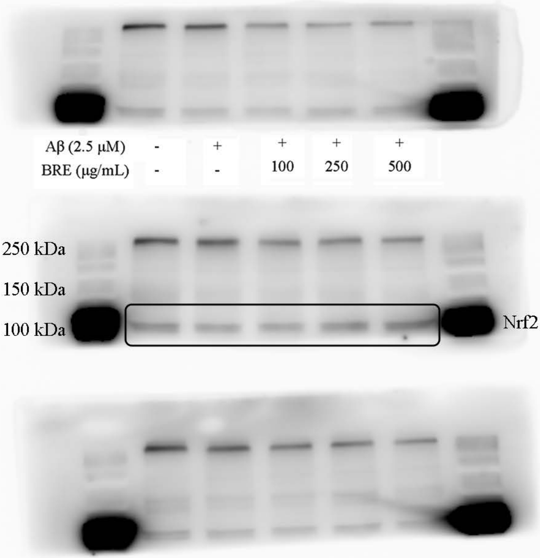
Aβ (2.5 µM) - + + + +
BRE (µg/mL) - - 100 250 500
250 kDa
150 kDa
100 kDa
Nrf2

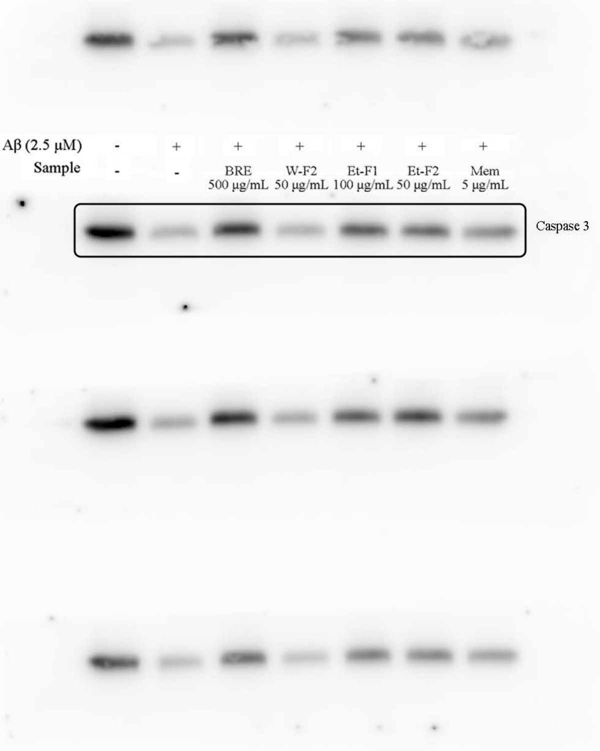
Aβ (2.5 μM) - + + + + + +
Sample - - BRE 500 μg/mL W-F2 50 μg/mL Et-F1 100 μg/mL Et-F2 50 μg/mL Mem 5 μg/mL
Caspase 3

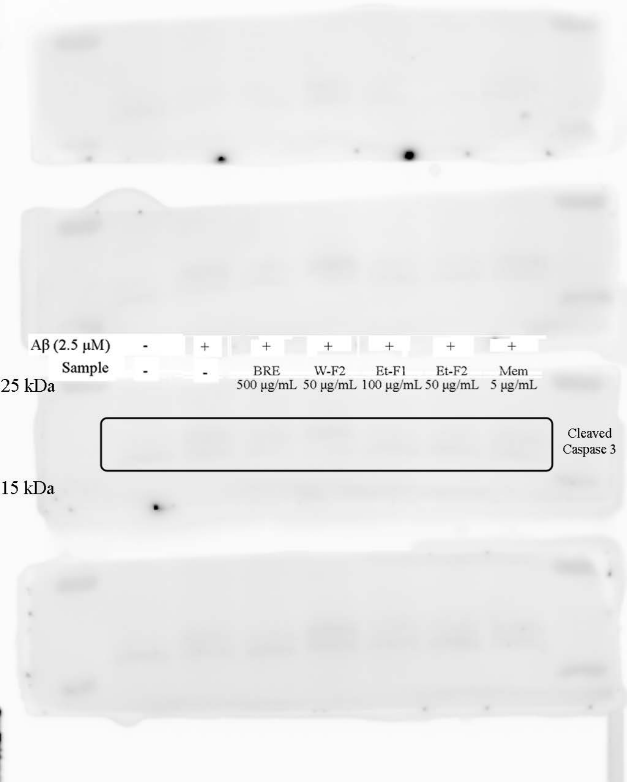
Aβ (2.5 µM) - + + + + + +
Sample - - BRE W-F2 Et-F1 Et-F2 Mem
500 µg/mL 50 µg/mL 100 µg/mL 50 µg/mL 5 µg/mL
25 kDa
15 kDa
Cleaved Caspase 3

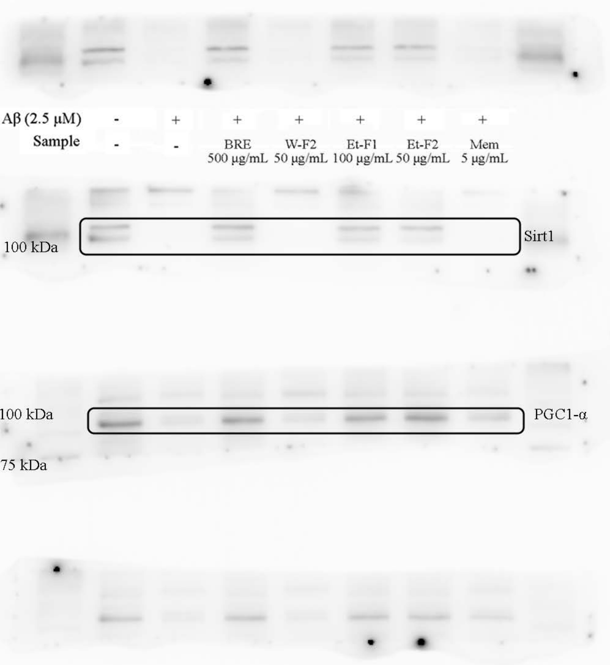

Aβ (2.5 μM)
-
+
+
+
+
+
+
Sample
-
-
BRE 500 μg/mL
W-F2 50 μg/mL
Et-F1 100 μg/mL
Et-F2 50 μg/mL
Mem 5 μg/mL
100 kDa
Sirt1
100 kDa
PGC1-α
75 kDa

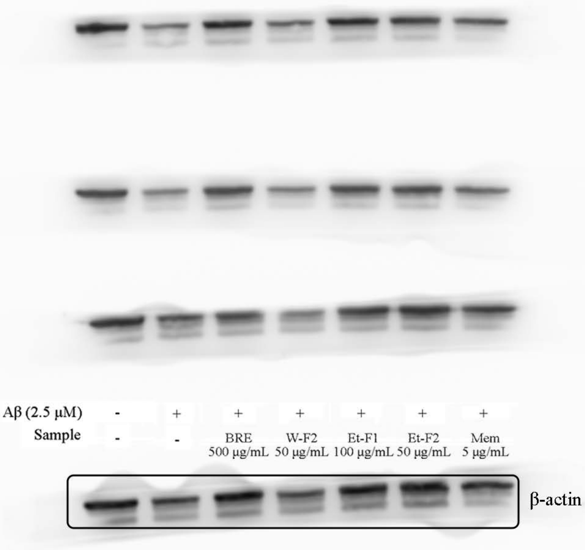
Aβ (2.5 μM)
-
+
+
+
+
+
+
Sample
-
-
BRE 500 μg/mL
W-F2 50 μg/mL
Et-F1 100 μg/mL
Et-F2 50 μg/mL
Mem 5 μg/mL
β-actin

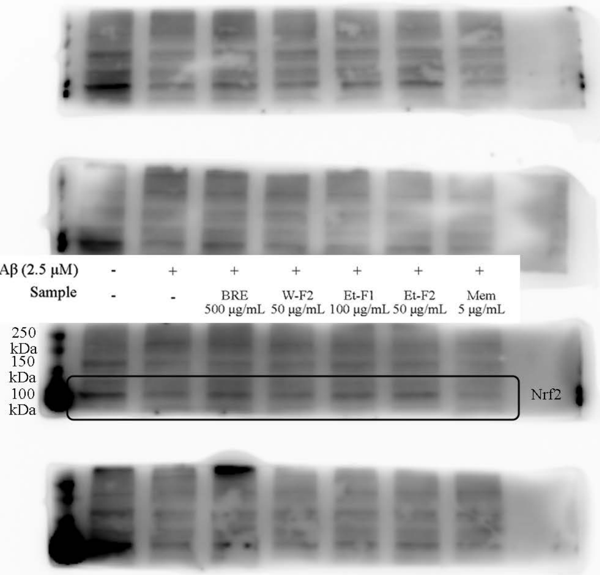
Aβ (2.5 μM) - + + + + + +
Sample - - BRE 500 μg/mL W-F2 50 μg/mL Et-F1 100 μg/mL Et-F2 50 μg/mL Mem 5 μg/mL
250 kDa
150 kDa
100 kDa
Nrf2

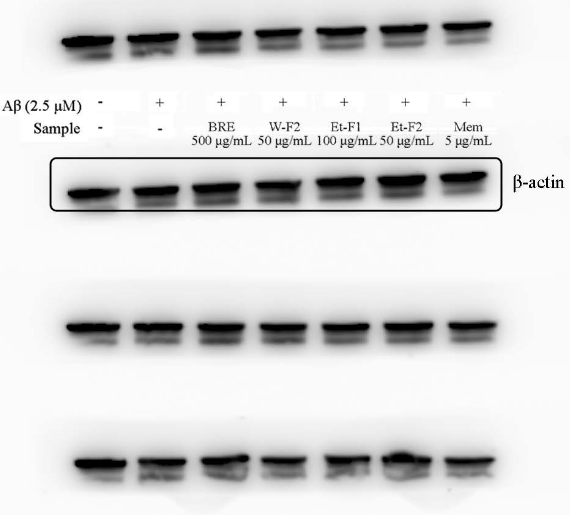

Aβ (2.5 μM) - + + + + + +
Sample - - BRE 500 μg/mL W-F2 50 μg/mL Et-F1 100 μg/mL Et-F2 50 μg/mL Mem 5 μg/mL
β-actin
